# A biosynthetic model of cytochrome *c* oxidase as an electrocatalyst for oxygen reduction

**DOI:** 10.1038/ncomms9467

**Published:** 2015-10-12

**Authors:** Sohini Mukherjee, Arnab Mukherjee, Ambika Bhagi-Damodaran, Manjistha Mukherjee, Yi Lu, Abhishek Dey

**Affiliations:** 1 Department of Inorganic Chemistry, Indian Association for the Cultivation of Science, 2A&2B Raja SC Mullick Road, Jadavpur Kolkata 700032, India; 2 Department of Chemistry, University of Illinois at Urbana-Champaign, Champaign, Illinois 61801, USA

## Abstract

Creating an artificial functional mimic of the mitochondrial enzyme cytochrome *c* oxidase (C*c*O) has been a long-term goal of the scientific community as such a mimic will not only add to our fundamental understanding of how C*c*O works but may also pave the way for efficient electrocatalysts for oxygen reduction in hydrogen/oxygen fuel cells. Here we develop an electrocatalyst for reducing oxygen to water under ambient conditions. We use site-directed mutants of myoglobin, where both the distal Cu and the redox-active tyrosine residue present in C*c*O are modelled. *In situ* Raman spectroscopy shows that this catalyst features very fast electron transfer rates, facile oxygen binding and O–O bond lysis. An electron transfer shunt from the electrode circumvents the slow dissociation of a ferric hydroxide species, which slows down native C*c*O (bovine 500 s^−1^), allowing electrocatalytic oxygen reduction rates of 5,000 s^−1^ for these biosynthetic models.

## Abstract (web summary)

An artificial functional mimic of cytochrome *c* oxidase is a long term research goal for the development of oxygen reduction electrocatalysts. Here, the authors use site directed mutants of myoglobin to develop an electrocatalyst for reducing oxygen to water under ambient conditions.

## Graphical abstract

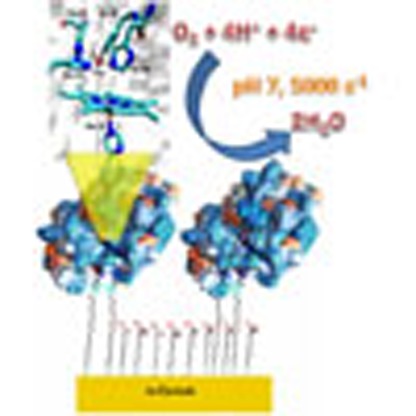

Mimicking the sophistication of naturally occurring enzymes has been a long-term goal of the scientific community. An artificial analogue that can perform equally well as its natural predecessor will not only provide deeper understanding of the native enzymes, but also enable the development of efficient artificial catalysts. For several decades now chemists have embarked on this daunting pursuit of emulating the efficiency and selectivity of naturally occurring enzymes and several important milestones have been achieved. Efforts from synthetic inorganic chemists have resulted in synthetic models of myoglobin (Mb), galactose oxidase, tyrosinase, cytochrome P450 and cytochrome *c* oxidase (C*c*O)^1^,^2^,^3^,^4^,^5^. Alternatively, there has been fervent pursuit of biochemical constructs inspired by natural metalloenzymes. A series of binuclear non-haem iron, cytochrome *c*, haem oxidases and iron–sulfur enzyme models have resulted from such efforts^4^,^6^,^7^,^8^,^9^,^10^,^11^,^12^. While none of the synthetic or biochemical models reported so far could match the reactivity exhibited by their natural counterparts, fundamental insights regarding the structure–function correlations of several metalloenzymes have been gained in the process^7^,^13^,^14^,^15^. In addition, key information about the secondary coordination sphere interactions present in the protein-active site, which play a dominating role in determining the electronic structure and reactivity of these metalloenzymes, have been identified^16^,^17^.

In a biosynthetic approach, stable naturally occurring proteins have been used as scaffolds for creating mimics of several metalloenzymes, such as hydrogenases which are involved in the reversible generation of H_2_ from water, haem proteins participating in electron transfer and O_2_-binding, non-haem iron and copper enzymes active in small molecule activation, and even novel enzymes containing non-native cofactors^18^,^19^,^20^,^21^,^22^,^23^,^24^. For example, using this approach, biosynthetic models that structurally and functionally mimic C*c*O and nitric oxide reductase have been reported^7^,^25^. Despite decades of focused effort, however, biosynthetic models with catalytic efficiencies approaching those of the naturally occurring metalloenzymes have remained elusive^26^,^27^,^28^. In this report, we communicate a biosynthetic model of C*c*O bearing the distal Cu_B_ and a tyrosine residue that is kinetically more competent in reducing O_2_ electrochemically than any known synthetic analogue, as well as native C*c*O itself.

X-ray crystallography of Mb and its mutant have revealed that its two propionate side chains project out of the protein surface into the solvent (Fig. 1)^7^. Taking advantage of this structural feature, we have previously developed an electrocatalytic O_2_ reduction system where the native haem cofactor in Mb is replaced by a modified hemin cofactor bearing an alkyne group (Hemin-yne, Fig. 2) so that electrons can be injected directly into the haem from a gold electrode to facilitate O_2_ reduction^29^. This method resulted in a Mb-functionalized electrode bearing 2.15 × 10^−12^ mol per cm^2^ of protein, which was characterized using several microscopic and spectroscopic techniques^29^. Over the last few years, a biosynthetic model of C*c*O has been reported in which two distal residues of Mb (L29 and F43) have been mutated to His, which along with the native His64, form a Cu-binding site, mimicking the distal Cu_B_-binding site present in C*c*O (Cu_B_Mb)^30^. Furthermore, in an attempt to mimic the conserved Tyr 244 residue in the C*c*O-active site, a G65Y mutant of Cu_B_Mb (G65YCu_B_Mb) containing redox-active tyrosine residue in the distal site and a variant where a tyrosine residue was crosslinked to the active site histidines were also created (Fig. 1)^31^.

Herein we report the electrocatalytic properties of the G65YCu_B_Mb (higher synthetic yields than the tyrosine crosslinked variant) immobilized on an Au electrode using the method developed for WT Mb^29^.

## Results

### Electrode characterization by SERRS

Surface-enhanced resonance Raman spectroscopy (SERRS) data (Fig. 3a) of the electrodes bearing the G65YCu_B_Mb protein with and without Cu_B_ show the oxidation and spin state marker *ν*_4_, *ν*_3_, *ν*_2_ and *ν*_10_ bands at 1,375 cm^−1^, 1,493 cm^−1^,1,585 cm^−1^ and 1,641 cm^−1^, respectively. The *ν*_4_, *ν*_3_ and *ν*_2_ values are consistent with the presence of a five-coordinated high spin haem in the active site on these electrodes bearing the biochemical constructs of C*c*O^32^. Also the bands at 1,504 cm^−1^ and *ν*_4_ at 1,641 cm^−1^ suggest the presence of a mixture of six-coordinate low spin species, which likely has H_2_O as the axial ligand. The positions of these bands in the G65YCu_B_Mb and their relative intensities are different from Hemin-yne (Supplementary Fig. 1)^29^.

### Electrode characterization by X-ray photoelectron spectroscopy

X-ray photoelectron spectroscopic (XPS) data of a G65YCu_B_Mb-bound Au electrode clearly indicate the presence of Fe, Cu, C, N and O elements (Supplementary Fig. 2, Supplementary Table 1). The 3p_3/2_, 2p_3/2_ and 2p_1/2_ binding energy peak for the Fe^III^ of haem group appear at 56.5 eV, 709.4 eV and 722.4 eV, respectively^33^,^34^. The 2p_3/2_ and 2p_1/2_ binding energy peak for Cu^II^ in the distal site appear at 931.7 eV and 951.8 eV, respectively^35^. The N_1s_ peak is broad (Supplementary Fig. 2), as it contains several components due to the presence of amide, haem pyrroles and the triazole groups (resulting from the covalent attachment of Hemin-yne) on the surface^33^. Similarly, the C_1s_ peak (Supplementary Fig. 2) contains contributions from different types of C atoms (aromatic, aliphatic, haem and so on) on these protein-modified surfaces^36^.

### Electrode characterization by CV

**Figure 2:**
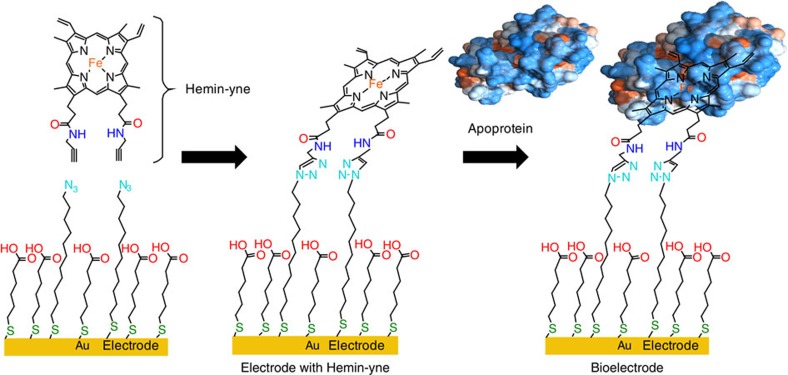
Construction of the electrode bearing the biosynthetic model. Reconstitution of apoprotein *in situ* with Hemin-yne groups that are covalently attached to mixed self-assembled monolayers of thiols on an Au electrode. The modified hemin is indicated as Hemin-yne.

**Figure 3:**
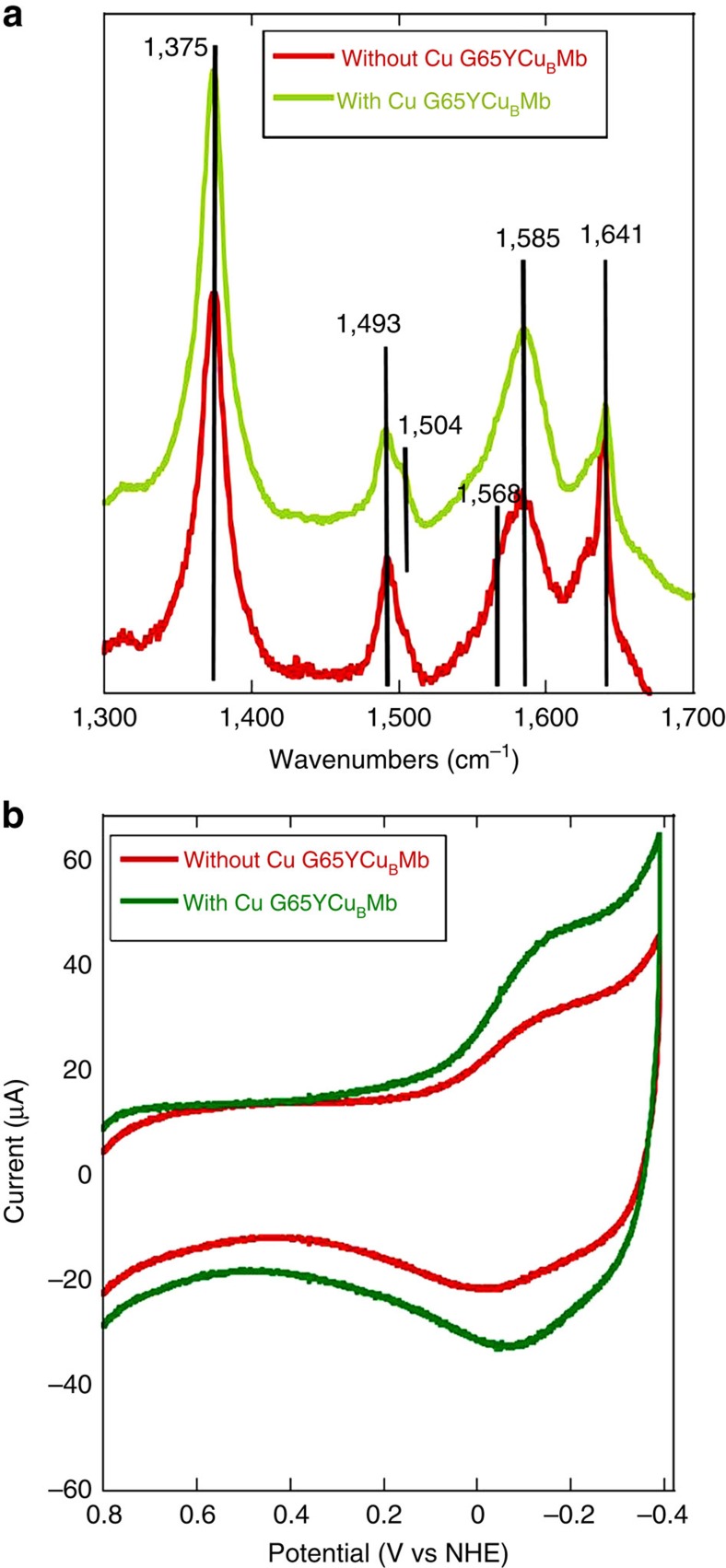
SERRS and CV data for the electrode fabricated with G65YCu_B_Mb mutant. (**a**) SERRS spectra of G65YCu_B_Mb with (green) and without (red) the distal Cu^2+^ in air-saturated 100 mM phosphate buffer (pH 7) solution. (**b**) Anaerobic CV of G65YCu_B_Mb without Cu_B_ (red) and after Cu_B_ binding (green). 2 V s^−1^ scan rate, in degassed, pH 7, 100 mM phosphate buffer using a Pt counter electrode.

Cyclic voltammetry (CV) of G65YCu_B_Mb with and without the distal Cu_B_ immobilized onto the electrodes in degassed buffer show the haem Fe^3+/2+^ midpoint reduction potential (*E*^1/2^) at −97 mV and −57.5 mV, respectively (Fig. 3b). The peak separation between the cathodic and the anodic peak for both the cases is ∼70 mV (ref. 37). Hemin-yne displays the Fe^3+/2+^ reduction potential at −70.0 mV in the absence of a protein and −135.0 mV when bound to wild-type apo Mb^29^. In the case of the G65YCu_B_Mb protein-bound electrodes, the Cu^2+/+^ process overlaps with the Hemin-yne Fe^3+/2+^ process, resulting in approximately twice the area under these CV peaks relative to the G65YCu_B_Mb-bearing electrodes prior to Cu_B_ loading. The integrated area under these CV features in the absence of Cu^2+^ indicates that there are 2.55±0.05 × 10^−12^ mol of protein per cm^2^ of the surface. The ratios of the integrated area under the CV features of G65YCu_B_Mb functionalized before and after loading the Cu_B_ is ∼1:2 (Table 1, fourth column) which is consistent with the expected 1:1 stoichiometry (that is, every G65YCu_B_Mb binds one Hemin-yne and one Cu^2+^ ion). Note that the *E*^1/2^ values of the hemin and Cu_B_ measured for these electrodes are slightly different from those estimated from potentiometric titration in solution^7^,^19^. This is likely due to the interfacial microenvironment of the –COOH-terminated Self Assembled Monolayer (SAM) which is known to shift the apparent formal potentials of redox-active species in its vicinity^38^. Thus the *in situ* reconstitution of the protein with Hemin-yne on the electrode is evident from the SERRS, XPS (Supplementary Fig. 2) and CV data (Supplementary Fig. 3, Supplementary Fig. 4). The presence of the Cu^2+^ at Cu_B_ site on the electrode is indicated by XPS and CV data. Taken together, these data indicate the assembly of the G65YCu_B_Mb, biosynthetic model of C*c*O covalently attached to the electrode via the linkage between the Hemin-yne and the azide terminated thiols created using click reaction (Fig. 2).

### O_2_ reduction reactivity of the electrode

**Figure 4:**
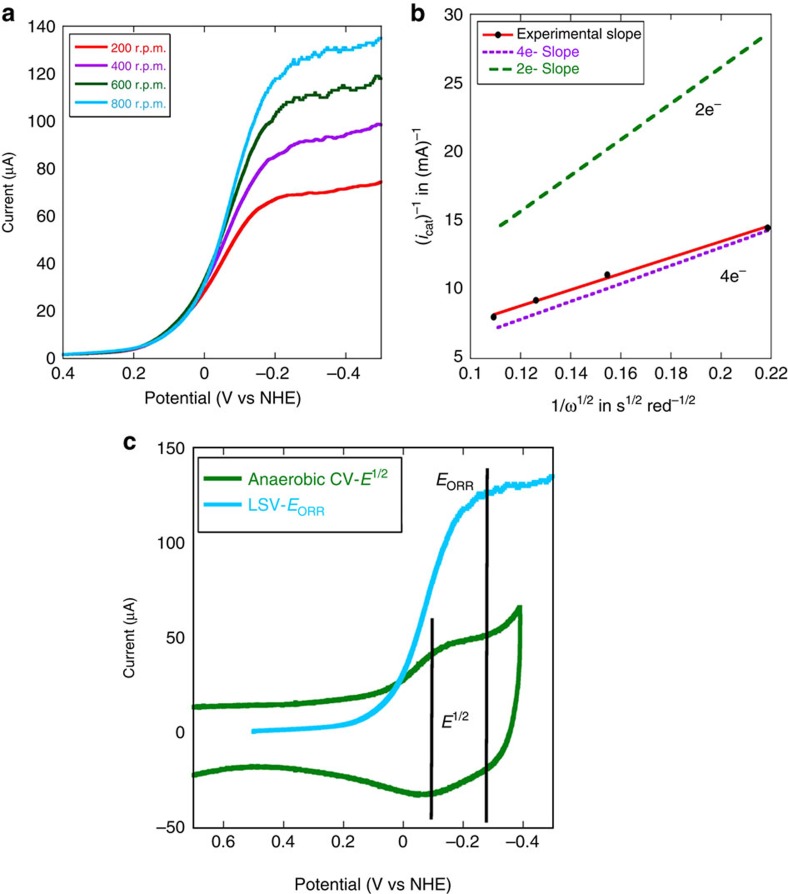
RDE data for electrode modified with G65YCu_B_Mb with Cu^2+^. (**a**) Linear sweep voltammogram of G65YCu_B_Mb with Cu^2+^ in air-saturated pH 7, 100 mM phosphate buffer solution at 100 mV s^−1^ scan rate, potentials are scaled relative to NHE and a Pt counter electrode is used. Data are collected at different rotation speeds (200 r.p.m.—red, 400 r.p.m.—purple, 600 r.p.m.—green, 800 r.p.m.—blue). (**b**) Plot of *i*_cat_^−1^ for G65YCu_B_Mb-bearing bioelectrode at −300 mV potential and at multiple rotation rates, with the inverse square root of the angular rotation rate (*ω*^−1/2^) (**c**) Difference between the potential for O_2_ reduction (*E*_ORR_) and the midpoint reduction potential of Fe^3+/2+^ redox couple (*E*^1/2^).

In linear sweep voltammetry experiments performed in aerated buffers, large electrocatalytic O_2_ reduction currents are observed by the G65YCu_B_Mb (with and without Cu^2+^) bearing bio-electrodes at pH 7 at room temperature, as the applied potential is lowered below +100 mV versus NHE (Fig. 4a, Supplementary Fig. 5). Thus as the potential of the electrode is lowered such that the iron in these proteins is reduced to Fe^II^, an electrocatalytic O_2_ reduction current is observed. It is important to note that the potential of O_2_ reduction reaction (ORR) (*E*_ORR_) is −263 mV, which is more negative than the *E*^1/2^(−97 mV), suggesting that the potential determining step of ORR is not the reduction of resting Fe^III^ to Fe^II^ but the reduction of a different species with −166 mV more negative potential (Fig. 4a inset). A more negative *E*_ORR_ relative to *E*^1/2^ is mechanistically significant (*vide infra*).

In these active sites, O_2_ may be reduced by 4e^−^ and 4H^+^ to H_2_O, or by fewer electrons to produce partially reduced oxygen species (PROS) like O_2_^−^and H_2_O_2_. The extent of 4e^−^ reduction and the second order rate constant (*k*_ORR_) of the ORR can be determined using rotating disc electrochemistry (RDE) where the catalytic O_2_ reduction current increases with increasing rotation rates (Fig. 4a,b) following the Kouteky–Levich (K–L) equation (equation (1))^39^.


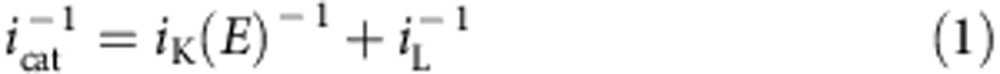


where, *i*_K_(*E*) is the potential-dependent kinetic current and *i*_L_ is the Levich current. *i*_L_ is expressed as


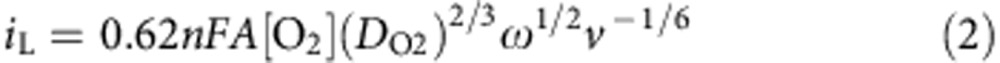


where *n* is the number of electrons transferred to the substrate, *A* is the macroscopic area of the disc (0.096 cm^2^), [O_2_] is the concentration of O_2_ in an air-saturated buffer (0.26 mM) at 25 °C, *D*_O2_ is the diffusion coefficient of O_2_ (1.8 × 10^−5^ cm^2^ s^−1^) at 25 °C, *ω* is the angular velocity of the disc and *ν* is the kinematic viscosity of the solution (0.009 cm^2^ s^−1^) at 25 °C (ref. 40).

Plot of *i*_cat_^−1^ at multiple rotation rates with the inverse square root of the angular rotation rate (*i*_cat_^−1^) for G65YCu_B_Mb (with Cu^2+^) (Fig. 4b) is linear. The slope of K–L plot is expressed as 1/[*n*{0.62*FA*(*D*_O2_)^2/3^*ν*^−1/6^}], which can be used to experimentally estimate the value of *n* where *n* is the number of electrons donated to the substrate, that is, O_2_. The slope obtained from the experimental data for G65YCu_B_Mb (Fig. 4b) is close to the theoretical slope (Fig. 4b, dotted purple line) expected for a 4e^−^ process and very different from the slope for a 2e^−^ process (Fig. 4b, dotted green line). Thus the G65YCu_B_Mb bioelectrode predominantly catalyses a 4e^−^/4H^+^ reduction of O_2_ to H_2_O at pH 7.

The intercept of the K–L plot is the inverse of the kinetic current (*i*_K_(*E*)^−1^), where *i*_K_(*E*) is expressed as^41^


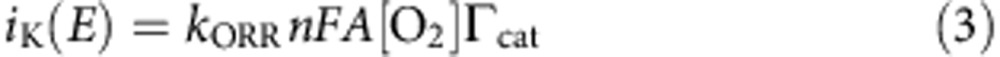


where, *n* is the number of electrons, *A* is the geometric surface area, [O_2_] is the bulk concentration of O_2_, Γ_cat_ is the surface coverage of the catalyst (obtained from the integration of the anaerobic CV data) and *k*_ORR_ is the second order rate constant for O_2_ reduction estimated at −300 mV. At this potential, in an oxygenated buffer, the G65YCu_B_Mb catalyst is involved in substrate diffusion-limited ORR. Using this equation (equation (3)) and the experimentally obtained *i*_K_(*E*) at −300 mV, the second order rate constant for O_2_ reduction G65YCu_B_Mb is evaluated to be 1.98 × 10^7^ M^−1^ s^−1^ (Table 2). The pseudo first order rate can be determined (Table 2) from the second order rate by taking into account the substrate, O_2_ concentration under these experimental conditions to be 0.26 mM (Supplementary Fig. 6). The catalytic ORR rate by G65YCu_B_Mb surpasses those reported for the best artificial synthetic analogues (Table 2).

The G65YCu_B_Mb biosynthetic Mb scaffold-based bio-electrode for O_2_ reduction is remarkably stable. Monolayers bearing covalently attached O_2_-reducing electrocatalysts reported so far have never been stable enough to allow these dynamic electrochemical experiments to determine the kinetic parameters (*k*_ORR_, number of electrons and so on). Enzymes like laccases, directly attached to chemically modified graphite electrodes, were found to be stable enough to be investigated with these hydrodynamic techniques^27^. The failure to perform these experiments has been attributed to degradation of the catalyst during RDE experiment, that is, very small turnover numbers presumably due to the production of PROS during ORR. Rotating ring disc electrochemistry (RRDE) shows formation of only ∼6% PROS by the G65YCu_B_Mb (Supplementary Fig. 7) during ORR, indicating that it reduces 96% of O_2_ to H_2_O consistent with the RDE data. During the RDE experiments (Fig. 4a), the G65YCu_B_Mb-functionalized electrodes bearing 10^−12^ mol of the catalyst reduced 1.8±0.3 × 10^−8^ mol of O_2_ (7±1 × 10^−3^ C total charge and 4e^−^ per O_2_ molecule) yielding a turnover number of at least 10^4^. The role of PROS in degrading the catalyst is established by the fact that the electrolytic current (at −0.3 V) remains stable in the presence of 50 μM catalase in solution (Supplementary Fig. 8).

## Discussion

**Figure 5:**
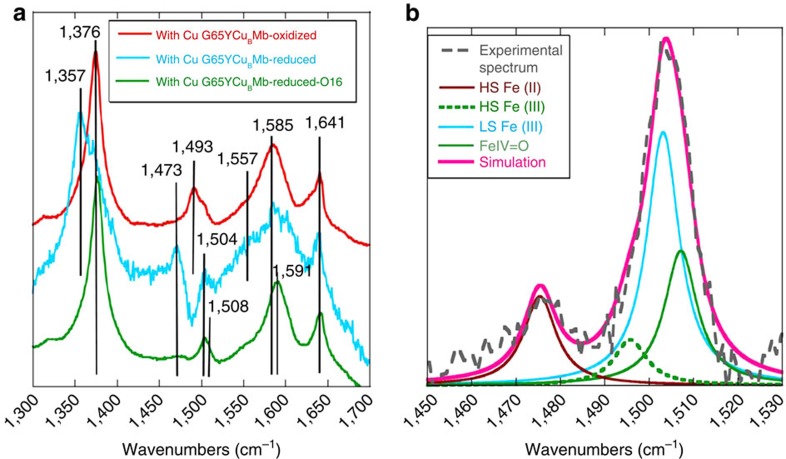
SERRS-RDE data of G65YCu_B_Mb-bearing electrode. (**a**) SERRS-RDE data of G65YCu_B_Mb-bearing electrode at oxidized (applied potential was 0 V with respect to Ag/AgCl reference electrode), reduced (applied potential was −0.4 V with respect to Ag/AgCl reference electrode) state and in the presence of O_2_ (O16) saturated 100 mM pH 7 phosphate buffer and (**b**) Components of the rR spectrum determined by simulating the spectra of G65YCu_B_Mb-bearing electrode in the presence of O_2_ (O16) saturated 100 mM pH 7 phosphate buffer indicated in green in **a**.

To understand the facile and selective O_2_ reduction catalysed by the G65YCu_B_Mb biochemical model, the recently developed SERRS-RDE technique is employed^42^. In this technique, the rR spectra of the catalyst (that is, G65YCu_B_Mb) bearing electrode is collected while the system is involved in steady state O_2_ reduction and the species accumulated in the steady state can be identified. For any species to accumulate in steady state, its rate of formation has to be greater than its rate of decay. Thus, while the species preceding the rate-determining step (rds) will accumulate at steady state, the accumulation of a species in steady state does not immediately imply its decay as the rds. In the absence of O_2,_ a high spin ferrous species is formed, characterized by a *ν*_4_ and *ν*_3_ vibrations at 1,357 cm^−1^ and 1,473 cm^−1^ (Fig. 5a, cyan), respectively, when a cathodic potential of −0.4 V is applied signifying reduction of the resting ferric state (Fig. 5a, red) to the active ferrous state at these potentials. However, when the same reducing potential is applied in an oxygenated buffer the SERRS-RDE data (Supplementary Fig. 9) clearly show the presence of different species during electrocatalytic ORR, which leads to broadening of the *ν*_4_, *ν*_3_ and *ν*_2_ regions (Fig. 5a, green) relative to the oxidized and reduced states (Fig. 5a, red and cyan). In particular, the *ν*_3_ and *ν*_2_ vibrations discernibly shift to higher energies as indicated by clear increase in intensities at 1,508 cm^−1^ and 1,591 cm^−1^ (Fig. 4b), suggesting the accumulation of Fe^IV^=O species during steady state ORR^43^. Signals from high spin ferrous, resting high spin ferric, low spin ferric and ferryl species with *ν*_3_ at 1,473 cm^−1^ (Fig. 5a, cyan; and Fig. 5b, brown), 1,493 cm^−1^ (Fig. 5a, red and Fig. 5b, dashed green), 1,504 cm^−1^ (Fig. 5a, cyan and Fig. 5b, cyan) and 1,508 cm^−1^ (Fig. 5a, green and Fig. 5b, green), respectively, could be convoluted by fitting the *ν*_3_ region of the spectrum. The lack of significant signal from the high spin ferrous species (weak *ν*_3_ at 1,473 cm^−1^) suggests that O_2_ binding to these species is facile in the steady state. These mutants use the basic design of Mb which has a very fast O_2_-binding rate (10^7^ M^−1^ s^−1^) (refs 44, 45). This rate is indeed ∼10 times faster than O_2_ binding to the haem a_3_ site of C*c*O (ref. 46). Similarly, the very weak intensity of the high spin (HS) Fe^III^ species indicates that the ET to Fe^III^ resting state is very facile at these potentials as may be expected due to direct attachment of the Hemin-yne to the electrode. The significant intensity of ferryl species (*ν*_2_ at 1,591 cm^−1^ and *ν*_3_ at 1,508 cm^−1^) entails the O–O bond cleavage leading to its formation to be faster than its decay via reduction under steady state. Thus the reduction of the resting Fe^III^ state, O_2_ binding to Fe^II^ are facile in G65YCu_B_Mb under the reaction conditions. The low spin ferric species accumulated during steady state ORR could be a dioxygen adduct or peroxide adduct similar to those observed in native C*c*O and its model systems^46^,^47^,^48^,^49^,^50^,^51^. The low frequency region shows ^18^O_2_-sensitive bands suggestive of the formation of a low spin ferric peroxide and Fe(IV)=O (Supplementary Fig. 10).

**Figure 6:**
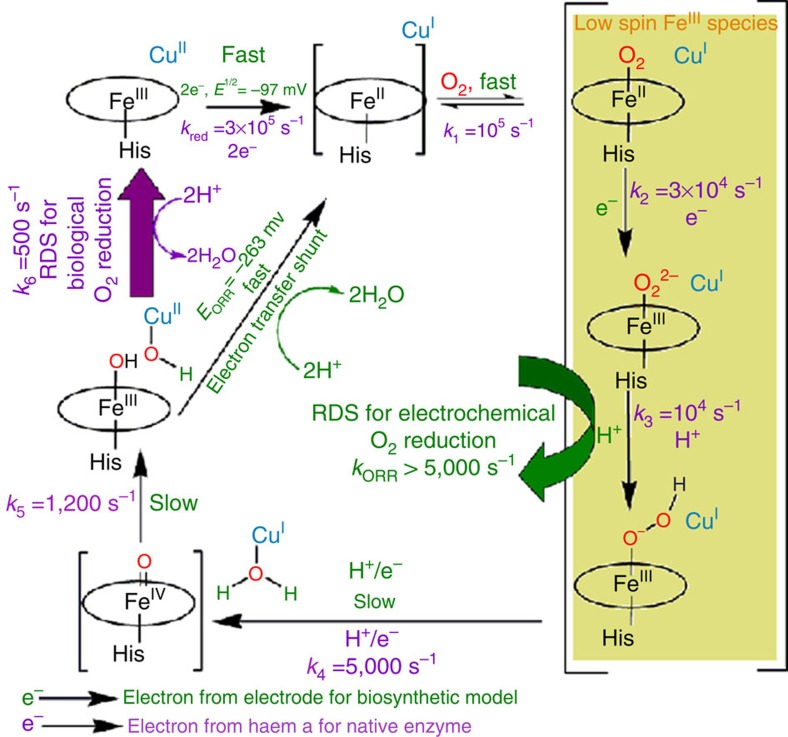
Mechanisms. Comparison between the mechanism of native C*c*O (ref. 46) in solution and the biosynthetic G65YCu_B_Mb model on electrode.

If one were to conceive of a Gedanken steady state turnover experiment with C*c*O where the electron transfer to the active site is very fast (that is, in the hypothetical situation where ET from Cyt *c* to C*c*O is not the rds) as the ET from the electrode to the active site is very fast due to direct attachment of the later to the former, the species that would accumulate during turnover, based on the Babkock–Wikström mechanism (Fig. 6, the parameters of native C*c*O are indicated in purple and G65YCu_B_Mb are indicated in green^52^ and the arrows in black indicate general route for O_2_ reduction by C*c*O followed in both the native system, as well as in the G65YCu_B_Mb-immobilized electrode) are the Fe^II^,Fe^II^-O_2_, Fe^III^-O_2_^2−^, Fe^III^-OOH, Fe^IV^=O and Fe^III^-OH species as the rates of formation of these species are greater than their rates of decay^53^,^54^. Out of these, the Fe^II^-O_2_, Fe^III^-O_2_^2−^ and Fe^III^-OOH species will have Raman signatures of low spin haem (Fig. 6), Fe^III^-OH will have rR signature of high spin haem and the Fe^IV^=O will have signatures unique to haem ferryl species^46^,^54^,^55^. The SERRS-RDE data show the presence of species having signatures of low spin Fe^III^ and Fe^IV^=O. While the later can originate from only a single species, the former can indicate the presence of any of the three species or a combination of them. The lack of significant high spin signal indicates that the biosynthetic model circumvents accumulation of Fe^III^-OH and resting Fe^III^ species in the steady state by facile ET. The overall rate-limiting step of native C*c*O in solution is the dissociation of hydroxide of the Fe^III^-OH end product of O_2_ reduction from haem to generate the active ferric resting form and has a first order rate constant of 500 s^−1^ (refs 46, 54, 55, 56). This dissociation is required during turnover as the potential of this hydroxide-bound form is likely to be more negative that the five-coordinate resting oxidized site (which will be regenerated after hydroxide dissociation) and will not be reduced by haem a. While the *E*^1/2^ of a haem a_3_ Fe^III^-OH species cannot be determined with confidence due to strong interaction potential and co-operativity between the haem a and haem a_3_ sites, the potential of formate and azide-bound high spin haem a_3_ site (analogous to hydroxide) is ∼130 to 200 mV more negative than the resting ferric site^57^,^58^. The potential determining step of ORR (defined as the electron transfer (ET) step in catalysis having lowest potential) by the G65YCu_B_Mb is 166 mV more negative than the *E*^1/2^ for the resting high spin ferric state and is likely to be the reduction of the Fe^III^-OH species. Thus the bioelectrode can circumvent the kinetic barrier associated with the dissociation of the hydroxide by directly reducing it to ferrous at 166 mV lower potential. This direct electron transfer to the ferric hydroxide species, which is an intermediate in the catalytic cycle of C*c*O, circumventing a slow step in catalysis, is an electron transfer shunt analogous to peroxide shunt in cytochrome P450, which overcomes the rate-determining O_2_ activation step of the native enzyme^59^. In the mass transfer controlled region of the catalytic current, all ET steps are facile and steps like O_2_ binding, protonation and O–O bond cleavage can be rds at these potential. The lack of HS Fe^II^ accumulation in the SERRS-RDE indicates that O_2_ binding to Fe^II^ is very fast and not the rds. The O–O bond cleavage in the Babkock–Wikström mechanism involves ET to the active site and not the rds as well. Thus rds of ORR by these biosynthetic models is likely to be the protonation of the Fe^III^-O_2_^−^ species with a first order rate of 5,000 s^−1^ (Fig. 6).

In an air-saturated buffer (0.26 mM O_2_), the pseudo first order rate constant of ORR by G65YCu_B_Mb is determined to be ∼5–6 × 10^3^ s^−1^ (*k*_ORR_ [O_2_]).The highest second order O_2_ reduction rate reported for any synthetic mimic of C*c*O is 1.2 × 10^5^ M^−1^ s^−1^; that too on a multilayer having 1,000 times more catalyst than the G65YCu_B_Mb electrodes^60^. The second order rate constant of G65YCu_B_Mb is 10^7^ M^−1^ s^−1^ which is, thus, 2 orders of magnitude higher than best synthetic haem/Cu-based O_2_ reduction electrocatalyst. Thus the selectivity and kinetic rate of the G65YCu_B_Mb-bearing electrode surpasses those reported for smaller synthetic analogues and illustrates the advantages of using a biochemical scaffold over a synthetic scaffold. Although the pseudo first order rate constant of the G65YCu_B_Mb is 10 times faster than the rate of native C*c*O in solution, such a comparison is vulnerable to differences in reaction conditions (for example, G65YCu_B_Mb is water soluble but C*c*O exists in membranes). Alternatively, erstwhile efforts resulting in electrodes bearing native C*c*O in a manner similar to these bio-electrodes show extremely sluggish O_2_ reduction^60^,^61^,^62^. This is due to improper alignment of this membrane-bound protein on the electrode, which precludes efficient electron transfer to the active site^63^,^64^,^65^. However, the direct attachment of haem to the electrode utilizing its solvent-exposed propionate groups (that is, a short circuit) enables fast electron transfer to the active site^29^. This is further supported by the fact that when ethynylferrocene (Fc) is attached to the same surface ∼25 mV peak separation is observed even at 5 V s^−1^ (Supplementary Fig. 11)^38^, suggesting that the ET is indeed fast. As a result when a C*c*O-functionalized SAM-covered Au electrode produces <1 μA electrochemical O_2_ reduction current at −300 mV, this bio-electrode produce ∼100 μA current at similar potentials^66^.

Finally, the G65YCu_B_Mb mutant has residues in the distal pocket that can help both electron and proton transfer during O_2_ reduction (Y65 in G65YCu_B_Mb). In C*c*O, the involvement of Tyr 244 residues in proton/electron transfer during O_2_ reduction is now widely accepted^67^,^68^. Previous biochemical and structural studies on these mutants had indeed indicated the close proximity of this residue to the distal site^7^,^19^,^30^. An analogous biochemical model without the Y65 residue, Cu_B_Mb, is not as stable as the G65YCu_B_Mb as the former degrades rapidly during the RDE experiments (Supplementary Fig. 12). In summary, a electron transfer shunt which circumvents the rate-determining dissociation of a ferric hydroxide species by directly reducing it at slightly negative potential, fast O_2_ binding, fast electron transfer to the active site and the presence of a protective Y65 residue in a biochemical model of C*c*O results in O_2_ reduction activity 100 times faster than the best synthetic models, order of magnitude faster than C*c*O immobilized on electrode and follows a mechanism comparable to that of native C*c*O in solution.

## Methods

### Materials

1-Azidoundecane-11-thiol and Hemin-yne were synthesized following the reported procedure^29^,^69^. 6-Mercaptohexanoic acid was purchased from Sigma Aldrich. Di-sodium hydrogenphosphate dihydrate (Na_2_HPO_4_. 2H_2_O) was purchased from Merck. 2, 6-lutidine was purchased from Avra Synthesis Pvt. Ltd. These chemicals were used without further purification. Au wafers were purchased from Platypus Technologies (1,000 Å of Au on 50 Å of Ti adhesion layer on top of a Si(III) surface). Transparent Au wafers (100 Å of Au on 10 Å of Ti) were purchased from Phasis, Switzerland. Au and Ag discs for the RRDE and SERRS experiments, respectively, were purchased from Pine Instruments, USA. The Mb mutants were prepared as reported in the literature^7^,^30^. Analysis of the components of the rR spectrum was done by using Lorenztian line shape of peak fit software.

### Instrumentation

All electrochemical experiments were performed using a CH Instruments (model CHI710D Electrochemical Analyzer). Bipotentiostat, reference electrode and Teflon plate material evaluating cell (ALS, Japan; http://www.als-japan.com/1398.html) were purchased from CH Instruments. The RRDE set-up from Pine Research Instrumentation (E6 series ChangeDisk tips with AFE6M rotor) was used to obtain the RRDE data. The mutant Mb-functionalized or SAM-covered Au surface (disc of 0.1 cm^2^ area for RDE, RRDE and wafer of 0.45 cm^2^ area for CV) was always used as the working electrode. The XPS data were collected in a Omicron (model: 1712-62-11) spectrometer using a high-resolution monochromatic Al-Kα source at 1,486.7 eV under 15 kV voltage and 10 mA current maintaining a base pressure of 5 × 10^−10^ mbar. The binding energies were calibrated to the Ag 3d_5/2_ peak at 368.2 eV. The resonance Raman experiments were done in the Kr^+^ Laser (Sabre Innova, Model—SBRC-DBW-K) purchased from Coherent, and the data were collected using the Spectrograph (Model—Trivista 555) from Princeton Instruments.

### Formation of mixed SAM and covalent attachment of Hemin-yne to it

Mixed self-assembled monolayer of 1-azidoundecan-11-thiol and 6-mercaptohexanoic acid was formed on immersing the properly cleaned Au wafers or disks into the deposition solution containing 1-azidoundecan-11-thiol and 6-mercaptohexanoic acid in 10 ml of ethanol in a desired ratio (typically 1:49). The total thiol concentration of these deposition solutions were always maintained at 1 mM. On this SAM Hemin-yne was covalently attached using ‘Click' reaction^29^.

### Reconstitution of Apo-G65YCu_B_Mb mutants to G65YCu_B_Mb

For all the experiments on heterogeneous SAM surfaces, the Hemin-yne modified –COOH SAM surfaces were incubated with a 20 μM apoprotein (Apo-G65YCu_B_Mb) solution for 2 h. The supernatant solution was drained and the surface was cleaned with water. The presence of Cu^2+^ in the non-haem-binding site is confirmed by electron paramagnetic resonance (EPR) spectroscopy (Supplementary Fig. 13). The immobilization of the mutant is further confirmed by the absorption spectra and SERRS of the surface fabricated with those mutants (Supplementary Fig. 14, Supplementary Fig. 15).

### Cyclic voltammetry

The CV was performed using Au wafers sandwiched between two Teflon blocks of the Plate material evaluating cell. All electrochemical experiments were done in pH 7 phosphate buffer containing potassium hexafluorophosphate. Anaerobic cyclic voltammetric experiments were done by using degassed buffer (three cycles of freeze–pump–thaw). Ag/AgCl reference electrode and Pt counter electrode were used throughout all the electrochemical experiments except the case of anaerobic experiments where only Ag wire was used as the reference electrode.

The peak areas were estimated by integrating the anodic/cathodic peak of the anaerobic CV of the mutant Mb-functionalized SAM-covered Au surface, using the data acquisition software itself. A line collinear with the background is used to subtract the background. The estimated area has been further confirmed by subtracting the background current of a SAM-functionalized electrode (Supplementary Fig. 11) bearing ferrocene. Both these approaches provide the same estimate.

To ensure that the SAM surface is stable during the electrocatalytic investigations, disc bearing just the SAM was subjected to several rotations (200–1,000 r.p.m.) and its capacitive current was found not to change, indicating that the SAM is retained on the electrode during these dynamic electrochemistry experiments (Supplementary Fig. 16). SAM can also be damaged when the protein atop the SAM degrades during ORR due to the reactive oxygen species produced. When an unstable electrocatalyst (Hemin-yne) decayed there was a steady loss of ORR current, indicating degradation of the active site but the capacitive currents of the SAM were unaltered (Supplementary Fig. 17).

### SERRS and SERRS-RDE

The excitation wavelength used in the Resonance Raman experiments was 406.7 nm and the power applied to the sample was 10–15 mW. The spectrograph was calibrated against naphthalene. The Ag surfaces were roughened before SERRS experiments following literature protocols^70^. The SERRS-RDE set-up is described in ref. 42. The data for the oxidized state was obtained by holding the potential of the disc at 0 mV versus NHE, and the data during steady state ORR was obtained by holding the disc at −400 mV versus NHE and the disc was rotated at 300 r.p.m. Normally data were acquired over a period of 300 s.

## Additional information

**How to cite this article:** Mukherjee, S. *et al*. A biosynthetic model of cytochrome *c* oxidase as an electrocatalyst for oxygen reduction. *Nat. Commun.* 6:8467 doi: 10.1038/ncomms9467 (2015).

## Supplementary Material

Supplementary Information Supplementary Figures 1-17, Supplementary Table 1 and Supplementary Reference

## Figures and Tables

**Figure 1:**
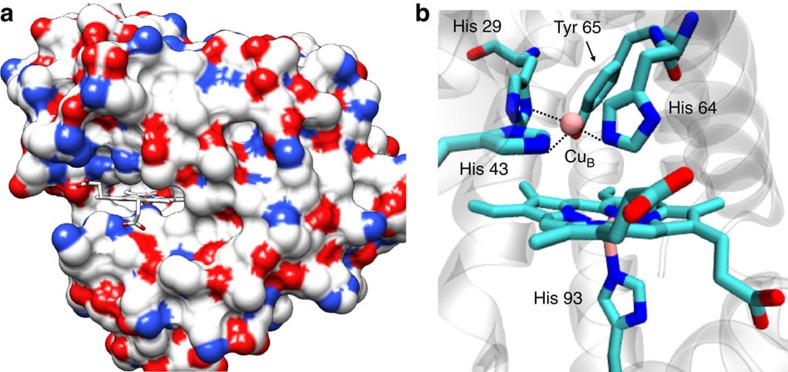
Crystal structure. Crystal structure of a Mb-based biosynthetic model of C*c*O, F33Y-Cu_B_Mb; pdb id: 4FWY. (**a**) The haem cofactor is in a cleft on the molecule protein surface, (colour coded according to the charge of the residues), with the propionate groups exposed to the solvent. (**b**) The computer model of G65YCu_B_Mb showing its catalytic centre containing the distal Cu_B_ bound to histidines and a tyrosine 65.

**Table 1:** *E* ^1/2^ and coverage.

Protein	Metal-binding sites	*E*^1/2^ (mV)	Integrated coverage (mol cm^−2^)
Hemin-yne reconstituted myoglobin^29^	Fe	−135.0±5.0	2.15±0.05 × 10^−12^
Hemin-yne reconstituted G65YCu_B_Mb	Fe	−57.5±5.0	2.55±0.05 × 10^−12^
	Cu_B_, Fe	−97.0±5.0	4.65±0.05 × 10^−12^

**Table 2:** *k*_ORR_ of different ORR catalysts.

ORR catalysts	Metals	*k*_ORR_	PROS (%)
G65YCu_B_Mb	Cu_B_, Haem	1.98 × 10^7^ M^−1^ s^−1^ or 5,148 s^−1^	∼6±1
Synthetic model^60^	Cu_B_, Haem	1. 2 × 10^5^ M^−1^ s^−1^	∼10±1

ORR, O_2_ reduction reaction; PROS, partially reduced oxygen species.

*k*_ORR_ determined at −0.4 V versus NHE; PROS determined at −0.12 V versus NHE in both cases.
