# Supplementary material for: A biosynthetic model of cytochrome c oxidase as an electrocatalyst for oxygen reduction
Source: Nat Commun. 2015 Oct 12;6:8467. doi: 10.1038/ncomms9467 (PMC4633646; doi:10.1038/ncomms9467)
Supplement: Supplementary Information — Supplementary Figures 1-17, Supplementary Table 1 and Supplementary Reference [file ncomms9467-s1.pdf]

# Supplementary Information

**SERRS of electrodes bearing Hemin-yne and myoglobin reconstituted with Hemin-yne**

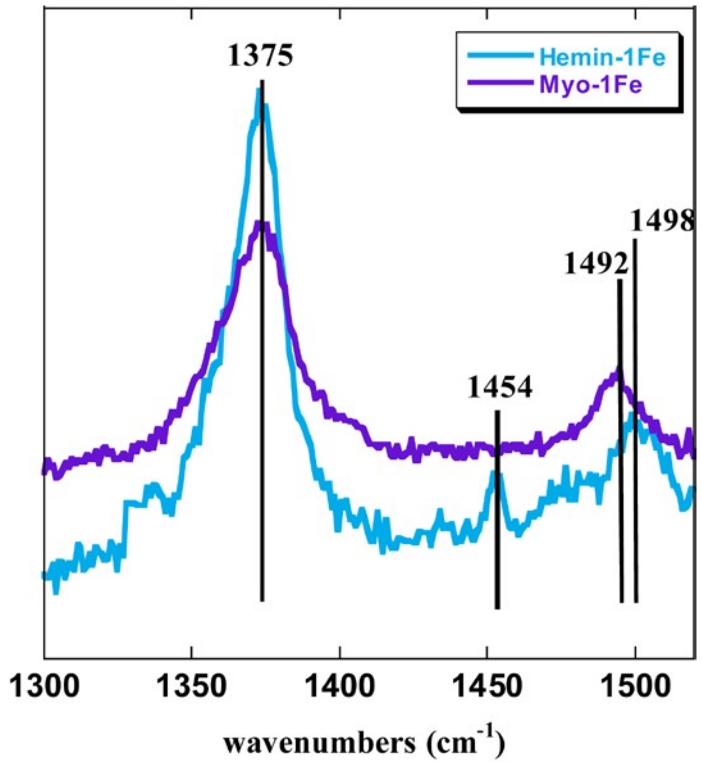


**Supplementary Figure 1.** SERRS spectra of Hemin-yne and myoglobin reconstituted with Hemin-yne in air saturated 100 mM phosphate buffer (pH 7) solution.

## X-ray Photoelectron Spectroscopic data

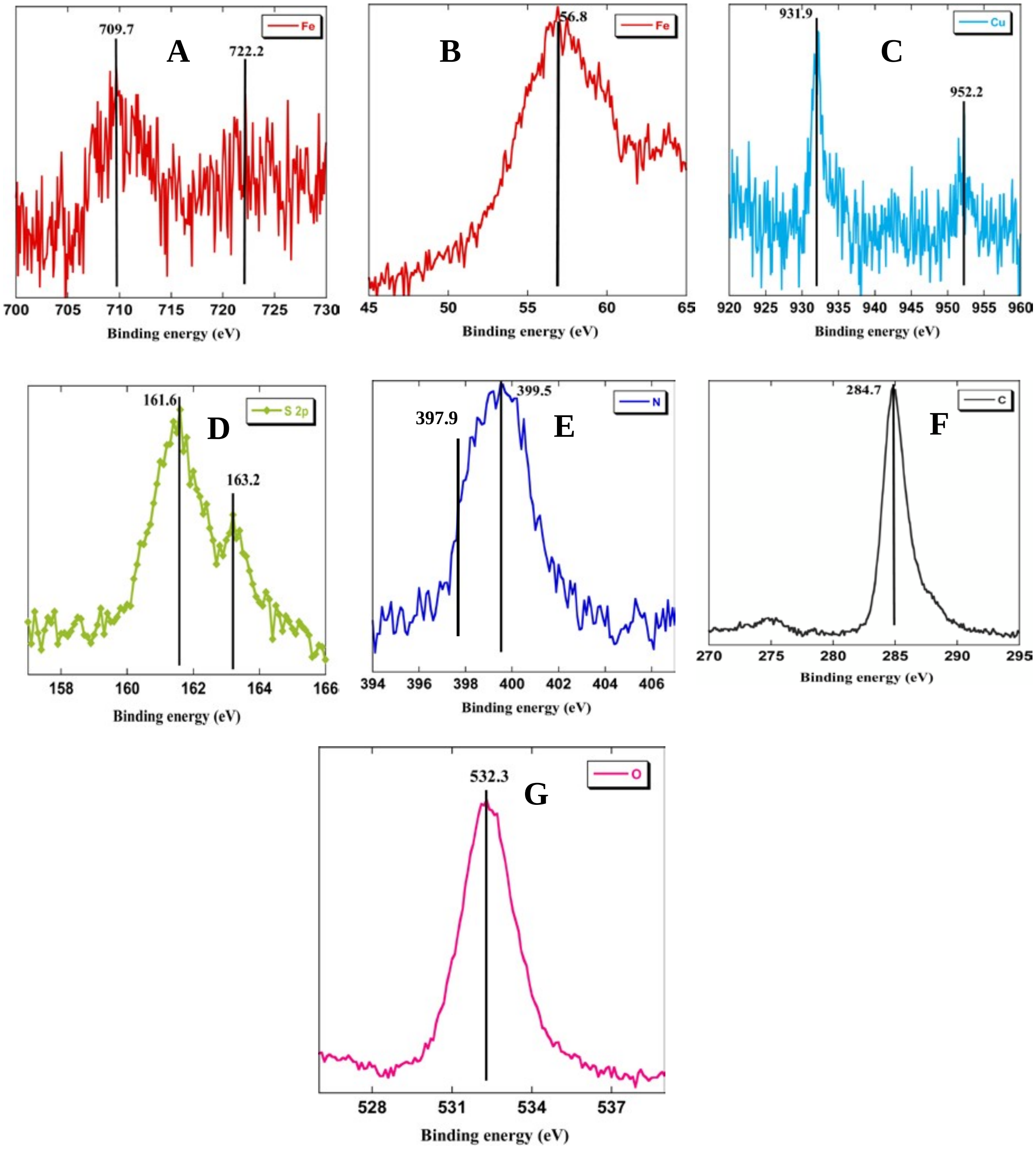


**Supplementary Figure 2.** High-resolution XP spectra for G65YCu$_B$Mb (with Cu) mutant immobilized on the SAM covered Au Surface using click reaction. (A) Fe 2p$_{3/2}$

and 2p$_{1/2}$ (B) Fe 3p$_{3/2}$ (C) Cu 2p$_{3/2}$ and 2p$_{1/2}$ (D) S 2p$_{3/2}$ and 2p$_{1/2}$ (E) N 1s$_{1/2}$ for triazole linkage (F) C 1s$_{1/2}$ (G) O 1s$_{1/2}$.

## Background subtraction from the CV of the electrode functionalized with G65YCu$_B$Mb protein

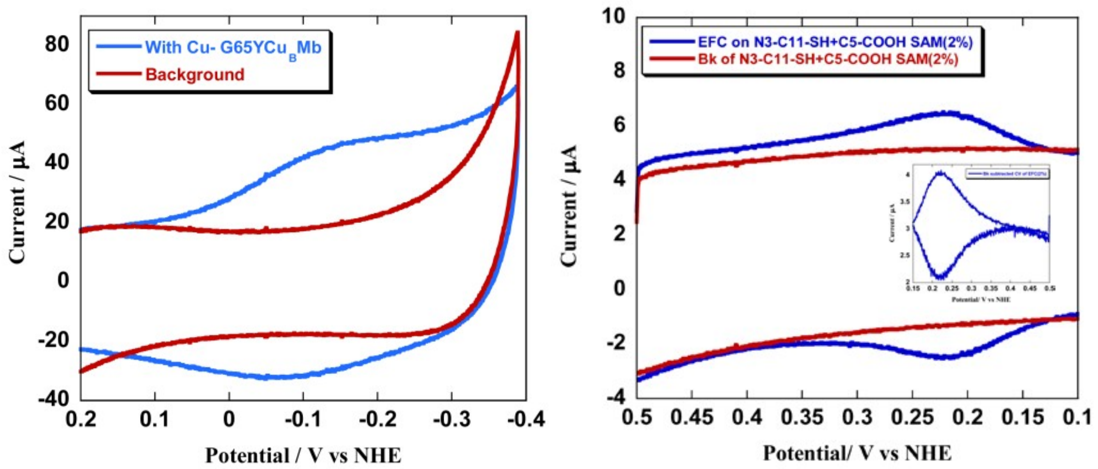


**Supplementary Figure 3. (left)** Overlay of the CV of the bioelectrode with the background current obtained before protein attachment **(right)** overlay of the background current with ethynylferrocene attached to the surface having same density of azide instead of the heimin-1Fe.

**$O_2$ Reduction by the Hemin-yne modified surface before and after G65YCu$_B$Mb (with Cu) protein binding**

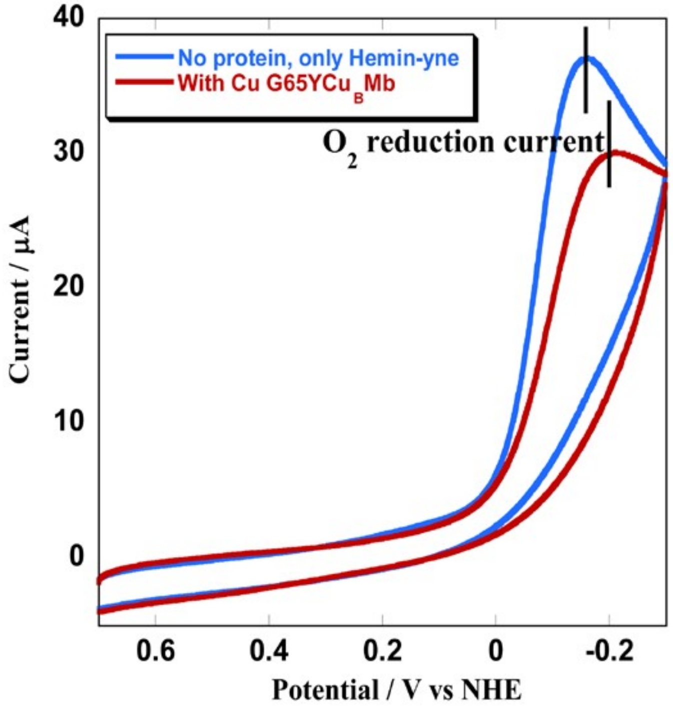


**Supplementary Figure 4:** Cyclic voltammogram for the $O_2$ reduction by the Hemin-yne modified surface before (blue) and after G65YCu$_B$Mb (with Cu) protein (red) binding, in 100 mM pH 7 phosphate buffer (air saturated) using Ag/AgCl electrode as reference and Pt as counter electrode.

**RDE data**

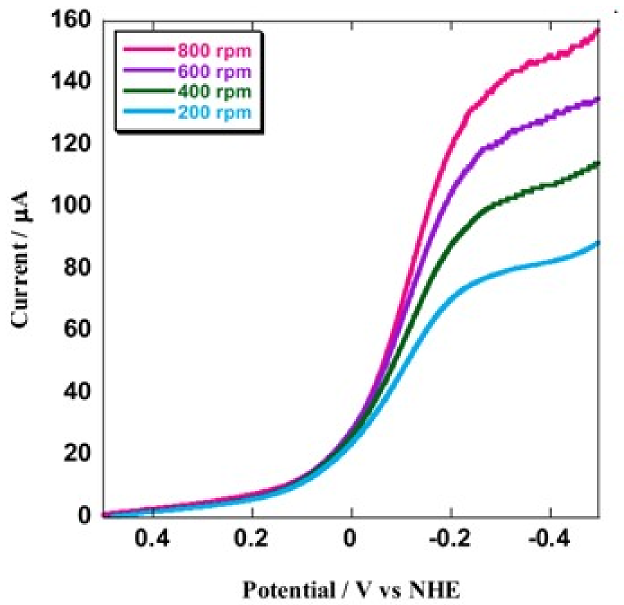


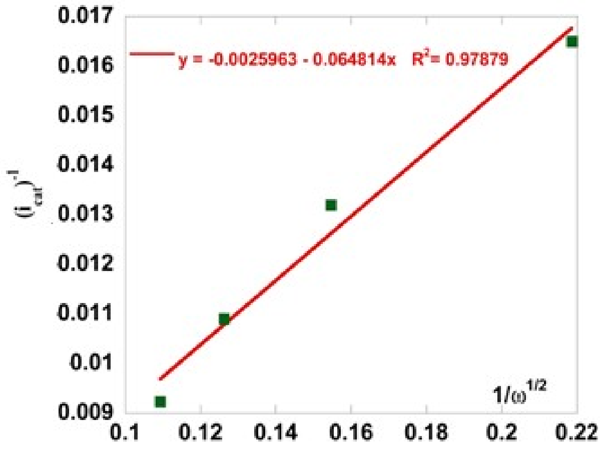


**Supplementary Figure 5:** Linear sweep voltammogram of G65YCu$_B$Mb (without Cu) modified electrode in air saturated pH7 100 mM phosphate buffer solution at 100 mV/s scan rate and using Ag/AgCl electrode as reference and Pt as counter electrode are plotted at different rotation speed (top). Plot of $(i_{cat})^{-1}$ at multiple rotation rates with the inverse square root of the angular rotation rate ($\omega^{-1/2}$) is also shown (bottom).

**LSV for electrocatalytic ORR at different concentration of $O_2$**

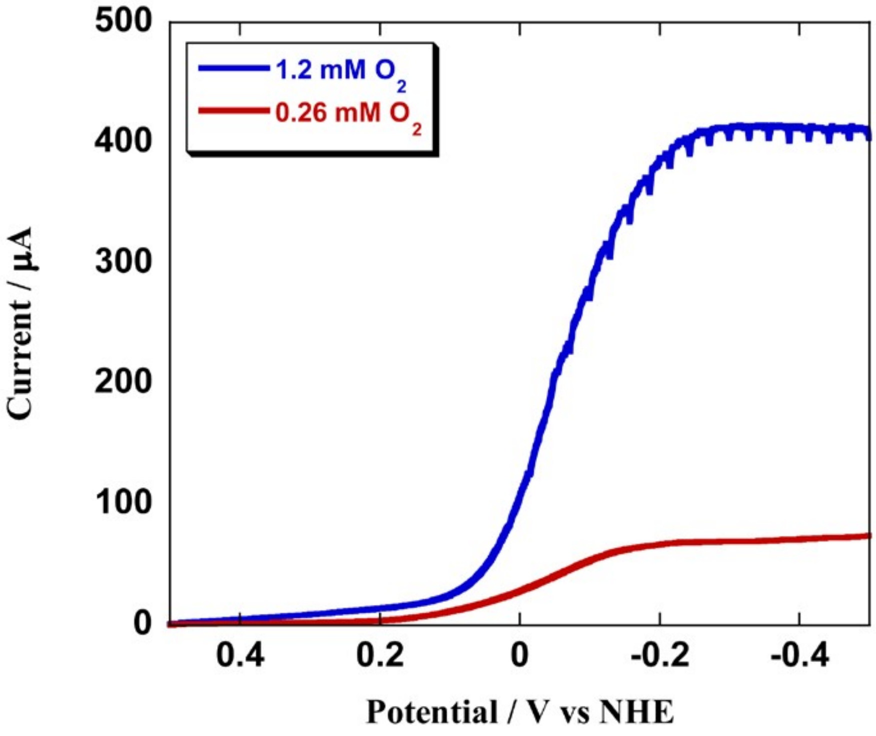


**Supplementary Figure 6:** LSV for electrocatalytic ORR at different concentration of $O_2$ at 300 rpm rotation and at 100 mV/s scan rate.

**RRDE**

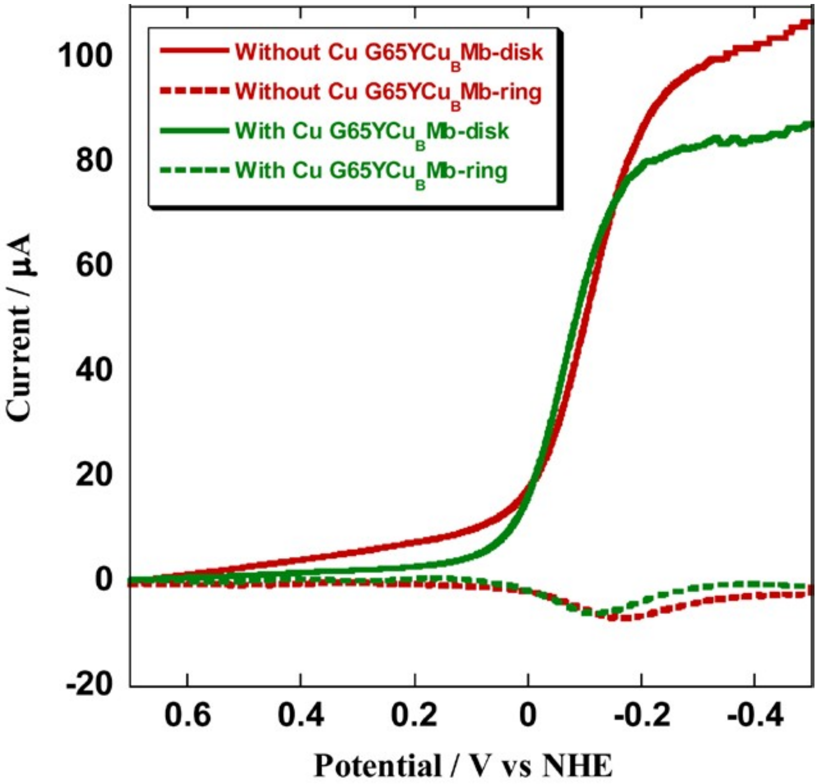


**Supplementary Figure 7:** Rotating ring disk electrode (RRDE) data of without Cu, G65YCu$_B$Mb (red) and with Cu, G65YCu$_B$Mb (green) at 10 mV/s and at 300 rpm rotation speed in air saturated pH 7 100 mM phosphate buffer using Ag/AgCl electrode as reference and Pt as counter electrode. The Pt current of the data for without Cu, G65YCu$_B$Mb (red) and with Cu, G65YCu$_B$Mb (green) were scaled by a factor of 10 and 15, respectively, for clear presentation.

**Degradation of With Cu G65YCu$_B$Mb modified surface on PROS production (both in presence and in absence of catalase)**

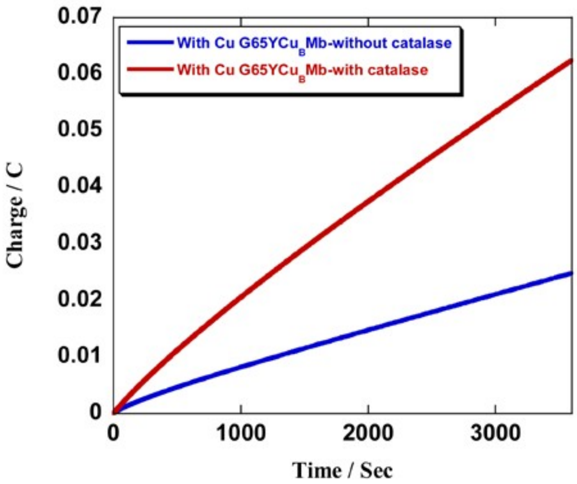


**Supplementary Figure 8:** Charge vs time plot for the degradation of with Cu G65YCu$_B$Mb modified surface with PROS production (in absence and in presence of 50 µM catalase in pH 7 phosphate buffer), at 200 rpm rotation and at -0.3 V vs NHE.

**Stability of the G65YCu$_B$Mb (with Cu) protein modified surface during electrolysis at -0.4 V vs NHE**

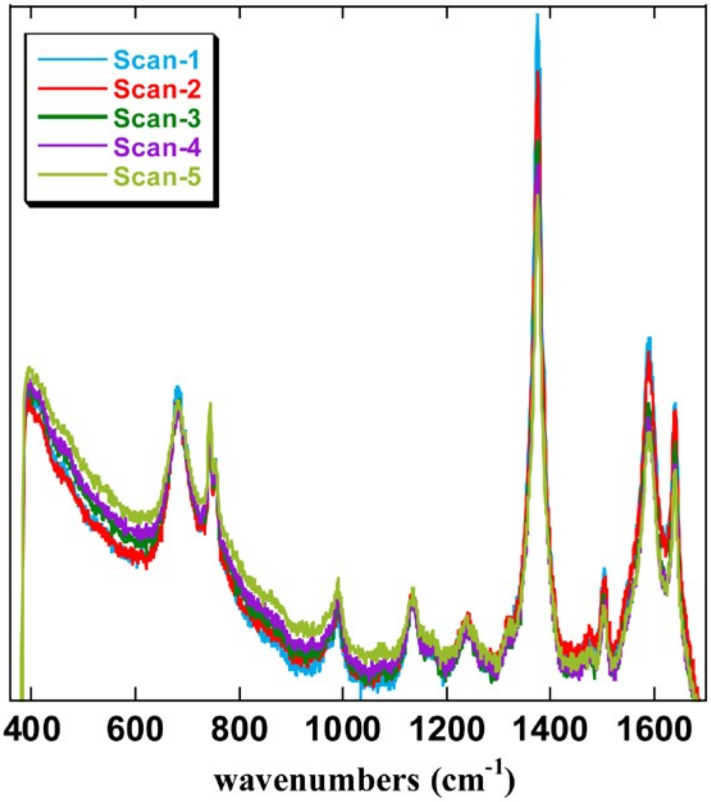


**Supplementary Figure 9:** SERRS-RDE spectra of G65YCu$_B$Mb (with Cu) protein modified surface, collected over 500 seconds during a bulk electrolysis experiment at -0.3 V vs NHE during which the electrode is under continuous rotation (300 rpm). Five scans are performed for 100 seconds each (one LSV is typically 20 seconds), in 100 mM pH 7 phosphate buffer saturated with $O^{18}$ isotope, using Ag/AgCl refrence electrode and Pt counter electrode.

**SERRS-RDE data obtained in $^{18}O_2$ and $^{16}O_2$ buffer.**

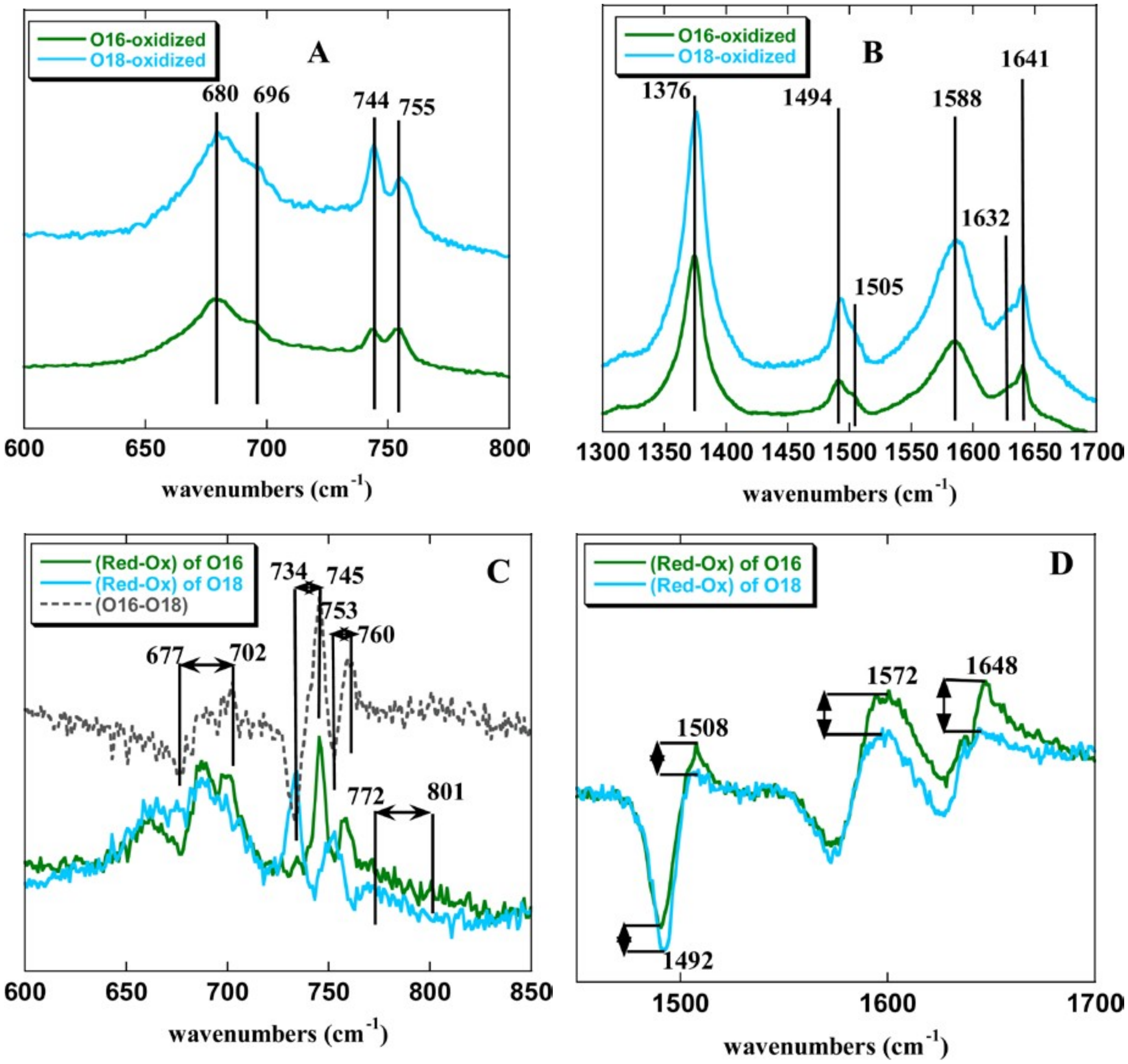


**Supplementary Figure 10:** SERRS-RDE on G65YCu$_B$Mb with Cu, immobilized bioelectrode in the presence of $O_2^{16}$ and $O_2^{18}$ saturated pH 7 phosphate buffer. (A) Oxidized spectra in $O_2^{16}$ and $O_2^{18}$ saturated pH 7 phosphate buffer at low range of wavenumbers (B) Oxidized spectra in $O_2^{16}$ and $O_2^{18}$ saturated pH 7 phosphate buffer at high range of wavenumbers (C) Difference spectra of reduced and oxidized state in $O_2^{16}$ and $O_2^{18}$ saturated pH 7 phosphate buffer at low range of wavenumbers, showing the

isotopic shift (D) Difference spectra of reduced and oxidized state in $O_2^{16}$ and $O_2^{18}$ saturated pH 7 phosphate buffer at high range of wavenumbers, showing the increase in intensity of the peaks from $O_2^{18}$ to $O_2^{16}$.

**CV of ethynylferrocene attached to the surface modified with the SAM of 1-azidoundecan-11-thiol and 6-mercaptohexanoic acid**

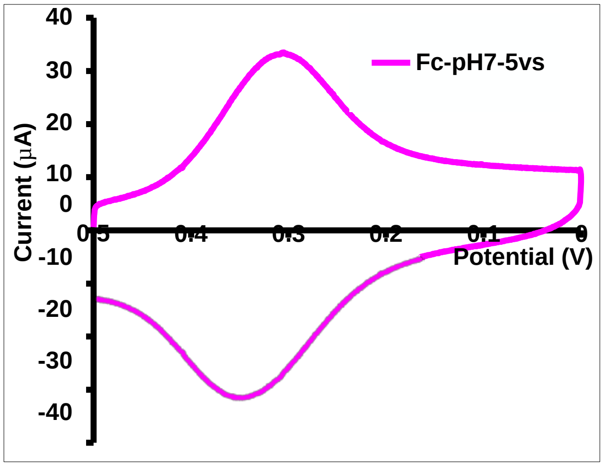


**Supplementary Figure 11.** CV of ethynylferrocene attached to the surface modified with the SAM of 1-azidoundecan-11-thiol and 6-mercaptohexanoic acid, at 5 V/s scan rate and using Ag/AgCl as the reference electrode and Pt as the counter electrode.

**Decay of Hemin-yne, Hemin-yne bound $Cu_BMb$ (no Y65)**

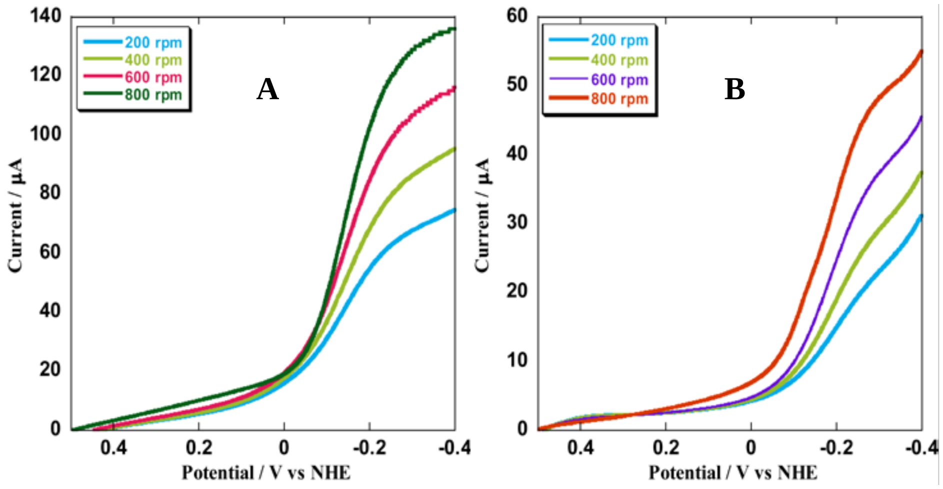


**Supplementary Figure 12:** Decay of $O_2$ reduction current at different angular rotation rate for (A) Hemin-yne (B) Hemin-yne bound Mb. Data clearly indicates the rapid decay of these catalysts.

**EPR**

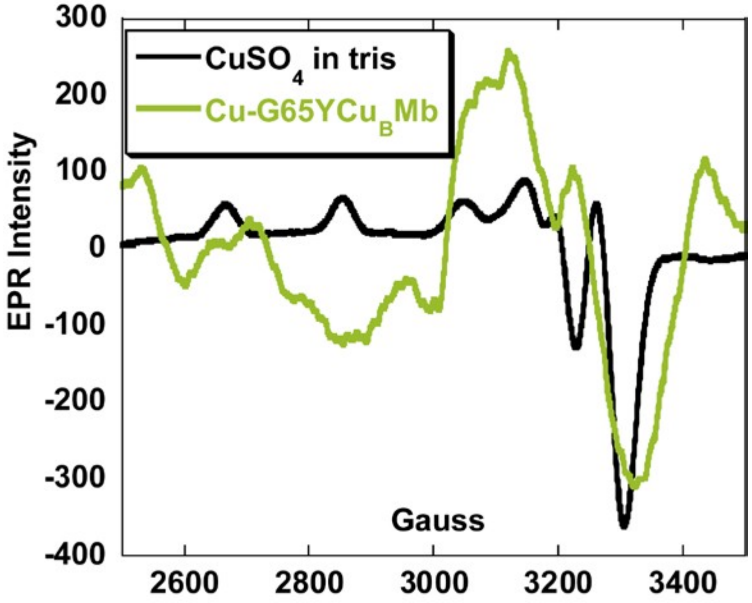


**Supplementary Figure 13:** EPR spectra of G65YCu$_B$Mb (green) with Cu compared with $CuSO_4$ (black) in 10 mM pH 7 phosphate buffer.

**UV-vis absorption spectra of G65YCu$_B$Mb with Cu on the Au surface**

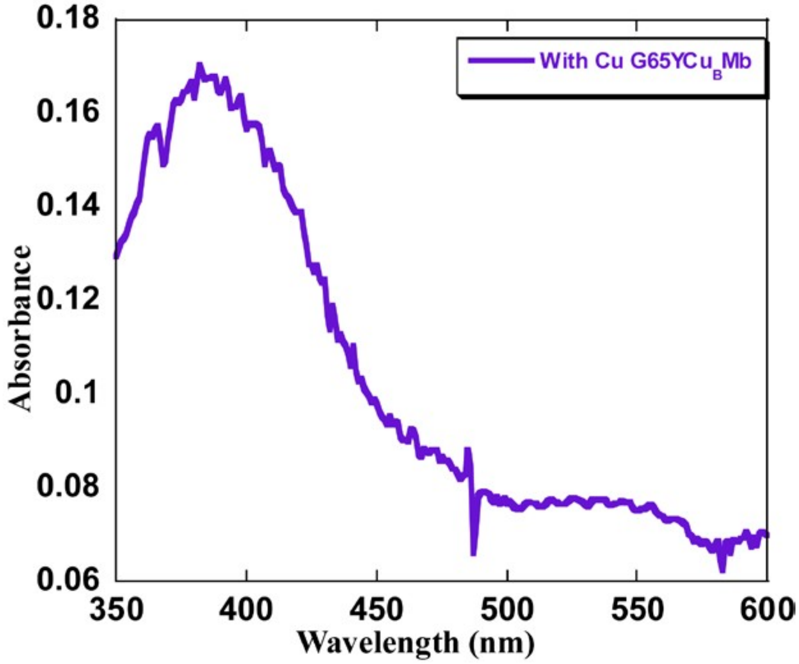


**Supplementary Figure 14:** Uv-vis absorption spectra of G65YCu$_B$Mb with Cu in 10mM pH 7 phosphate buffer.

**Fe-His stretch**

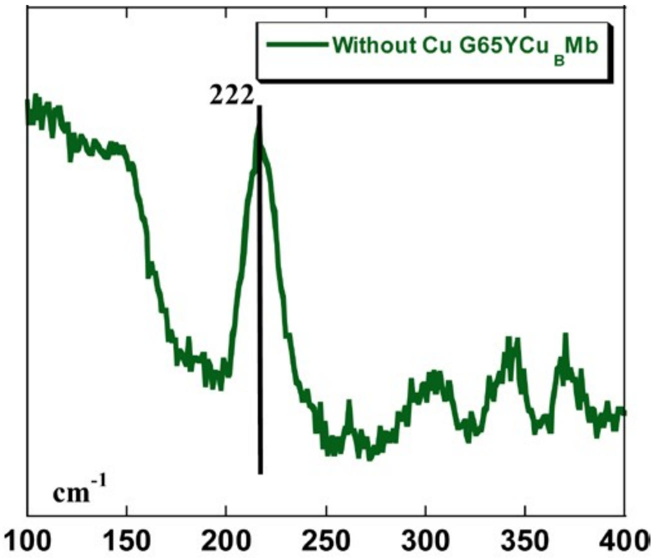


**Supplementary Figure 15:** Resonance Raman spectra of the homogenous solution of G65YCu$_B$Mb (green) with Cu indicating the Fe-His stretching frequency at 220 cm$^{-1}$.

**Stability of the mixed SAM of 1-azidoundecan-11-thiol and 6-mercaptohexanoic acid after rotations**

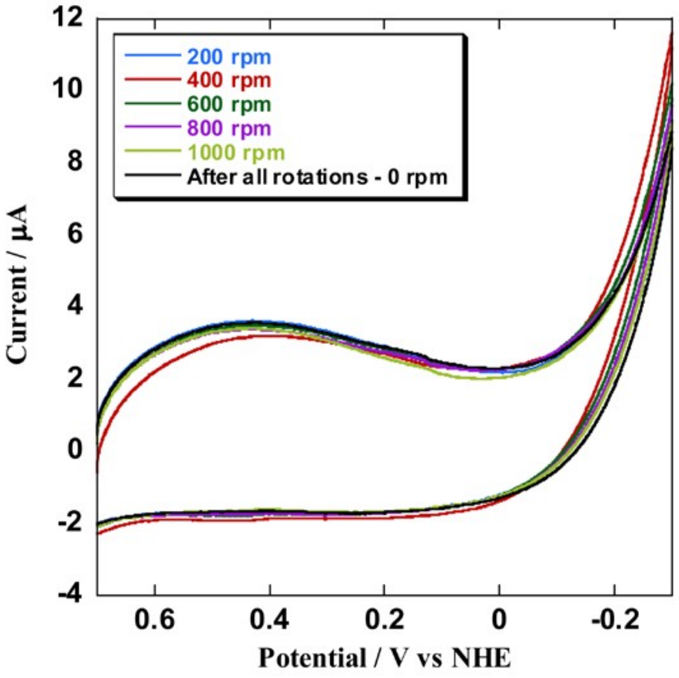


**Supplementary Figure 16:** Cyclic voltammogram of the background current of mixed SAM of 1-azidoundecan-11-thiol and 6-mercaptohexanoic acid, in 100 mM pH 7 phosphate buffer at after rotating the electrode at different rotation rates. (Ag/AgCl reference and Pt counter electrode)

**Stability of the $G65YCu_BMb$ (with Cu) protein modified surface after rotations**

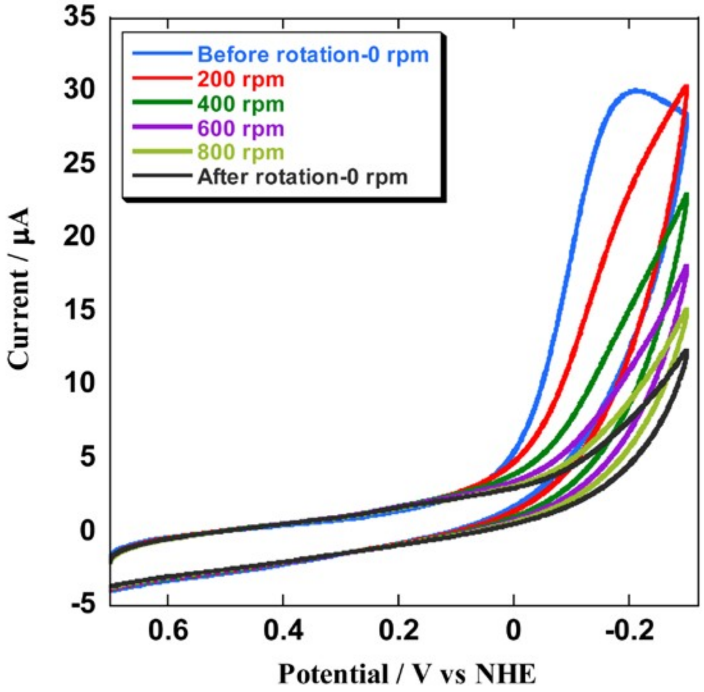


**Supplementary Figure 17:** Cyclic voltammogram of the $G65YCu_BMb$ (with Cu) protein modified surface, in 100 mM pH 7 phosphate buffer at different rotation rate using Ag/AgCl electrode as reference and Pt as counter electrode.

**Supplementary Table 1.** XPS data of G65YCu$_B$Mb (with Cu) modified Au electrode

| | | Binding Energies (eV) |
|---|---|---|
| **Fe** | $2p_{3/2}$ | 709.7 |
| | $2p_{1/2}$ | 722.2 |
| | $3p_{3/2}$ | 56.8 |
| **Cu** | $2p_{3/2}$ | 931.1 |
| | $2p_{1/2}$ | 952.2 |
| **S**[1] | $2p_{3/2}$ | 161.6 |
| | $2p_{1/2}$ | 163.7 |
| **N** | $1s_{1/2}$ | 397.9, 399.5 |
| **C** | $1s_{1/2}$ | 284.7, 287.8 |
| **O**[1] | $1s_{1/2}$ | 532.3 |

**Supplementary Reference**

1. Yuan, S. J.; Pehkonen, S. O., Surface characterization and corrosion behavior of 70/30 Cu-Ni alloy in pristine and sulfide-containing simulated seawater. *Corros. Sci.* **49**, (3), 1276-1304, (2007).
